# INTEGRATING AGE INCLUSIVITY WITH DEI EFFORTS ON AGE-FRIENDLY UNIVERSITY (AFU) CAMPUSES

**DOI:** 10.1093/geroni/igac059.1061

**Published:** 2022-12-20

**Authors:** Joann Montepare, Kimberly Farah, Peter Lichtenberg

## Abstract

The pioneering Age-Friendly University (AFU) initiative, endorsed by GSA’s Academy for Gerontology in Higher Education (AGHE), calls for institutions of higher education to respond to shifting demographics and the needs of age-diverse, older populations through more age-friendly programs, practices, and partnerships. Over 85 institutions in the United States, Canada, European countries, and beyond have joined the global network and endorsed the 10 AFU principles, with even more showing interest in becoming partners in the movement. One key foundational area identified by AFU research efforts and partners is integrating age inclusivity with ongoing diversity, equity, and inclusion (DEI) efforts on campuses. This symposium explores the need for this integration featuring AFU partners who will offer their observations and recommendations. Bowen and colleagues will open the session with data from their national study of age-friendliness in U.S. institutions to describe their insights regarding the state of age diversity on campuses and the experiences of students, faculty, and staff that call for greater age inclusivity. Morrow-Howell and colleagues will present data from interviews with DEI officers that identify institutional considerations for inclusion efforts. Andreoletti and colleagues will offer specific curricular and related strategies for connecting age-inclusivity efforts with DEI campus efforts. Gugliucci will discuss considerations regarding age-inclusive images and messages in health professions education including the inclusion of identifiable DEI objectives in syllabi. As discussant, GSA president Lichtenberg will comment on age-inclusivity efforts in higher education within GSA’s broader commitment to diversity, equity, and inclusion.

